# Preparation Prior to Coverage: A Biological Staging Concept for Predictable Root Coverage Using Amniotic Membrane

**DOI:** 10.7759/cureus.101949

**Published:** 2026-01-20

**Authors:** Charvi Kundu, Soundarya Singh, Athira Balagopal, Mayur Kaushik, Roopse Singh

**Affiliations:** 1 Department of Periodontology, Subharti Dental College, Swami Vivekanand Subharti University, Meerut, IND; 2 Department of Oral and Maxillofacial Surgery, Subharti Dental College, Swami Vivekanand Subharti University, Meerut, IND; 3 Department of Periodontology and Implantology, Subharti Dental College, Swami Vivekanand Subharti University, Meerut, IND

**Keywords:** amniotic membrane, gingival recession, keratinized tissue, lateral pedicle graft, periodontal plastic surgery, root coverage

## Abstract

Gingival recession presents a clinical challenge when keratinized tissue width is limited, even with adequate tissue thickness. This case report describes a staged, biologically driven approach to localized recession in relation to tooth 43. Keratinized tissue was first augmented using a freshly procured amniotic membrane, preserved in glycerol to maintain integrity and antimicrobial properties, treated with penicillin powder prior to surgery, and rinsed with sterile saline to ensure biocompatibility. After one month, a lateral pedicle graft was performed to cover the recession defect. Postoperative evaluation demonstrated a stable soft-tissue architecture with a satisfactory gingival contour. This case emphasizes the value of biologic recipient site preparation in enhancing the predictability and success of periodontal plastic surgery.

## Introduction

Gingival recession, defined as the apical migration of the gingival margin, is a common periodontal condition that can lead to root exposure, dentinal hypersensitivity, root caries, and esthetic concerns. Achieving predictable root coverage remains a central objective in periodontal plastic surgery; however, outcomes are influenced by the width and thickness of keratinized tissue. Adequate keratinized tissue not only supports long-term periodontal stability but also enhances the functional and esthetic success of root coverage procedures. In cases where keratinized tissue width is limited despite favorable tissue thickness, conventional root coverage techniques may produce suboptimal results or relapse [1].

Various techniques, such as free gingival grafts, subepithelial connective tissue grafts, and soft-tissue substitutes, have been used to increase the width of attached gingiva following such apical augmentation procedures.

Biologic adjuncts, such as amniotic membranes, have gained prominence in periodontal therapy due to their unique regenerative properties. These membranes contain growth factors, promote epithelialization, stimulate angiogenesis, modulate inflammation, and exhibit antimicrobial activity. Their use in soft tissue augmentation allows for predictable enhancement of keratinized tissue width and provides an optimized recipient site for subsequent surgical procedures. Biologic adjuncts such as injectable platelet‑rich fibrin and hyaluronic acid have also been investigated for gingival phenotype modification and soft‑tissue augmentation, showing variable but promising effects on keratinized tissue width and thickness [2].

The lateral pedicle graft is a well-established root coverage technique that utilizes adjacent keratinized tissue to cover the recession defect, ensuring vascularized flap transfer and favorable healing [3]. Combining biologic augmentation with this surgical approach allows for staged, controlled management of gingival recession, thereby maximizing clinical outcomes [4]. This report describes a case of localized gingival recession in relation to tooth 43 managed with amniotic membrane-assisted keratinized tissue augmentation followed by a lateral pedicle graft, highlighting the advantages of this integrated strategy [5].

## Case presentation

**Figure 1:**
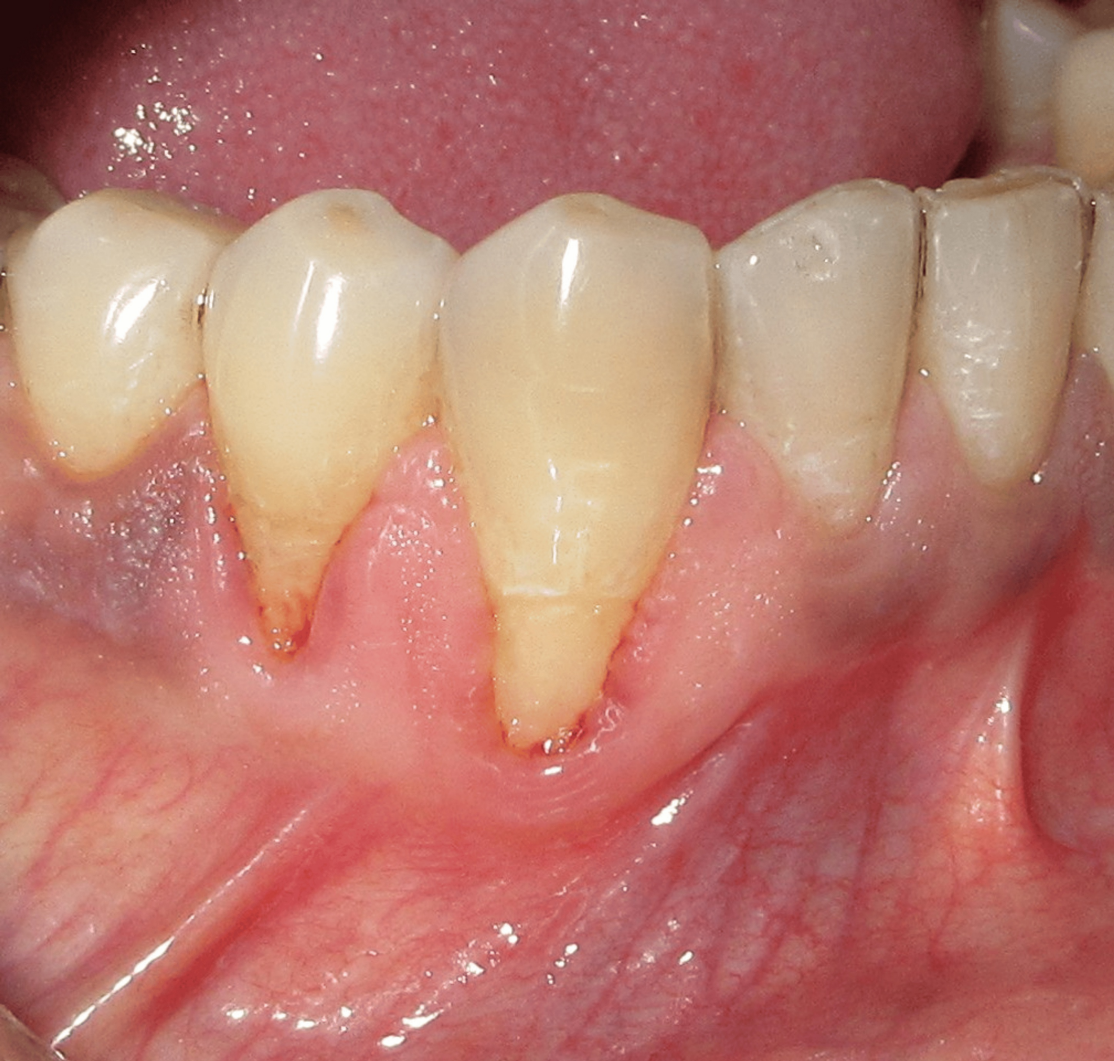
Preoperative recession in relation to tooth 43

A 28-year-old systemically healthy patient presented with localized gingival recession in relation to tooth 43 (Figure 1). Clinical examination revealed adequate gingival thickness but a limited width of keratinized tissue, which could compromise predictable root coverage. After discussing treatment options and obtaining informed consent, a staged, biologically driven approach was planned: initial augmentation of keratinized tissue using an amniotic membrane, followed by a lateral pedicle graft to cover the recession defect.

**Figure 2:**
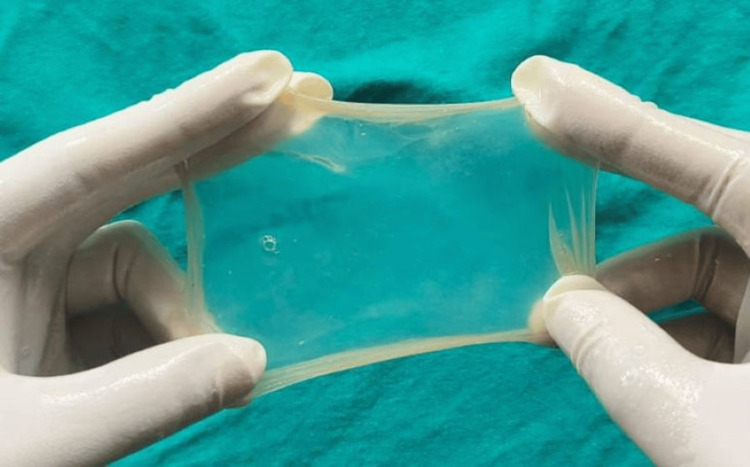
Amniotic membrane

**Figure 3:**
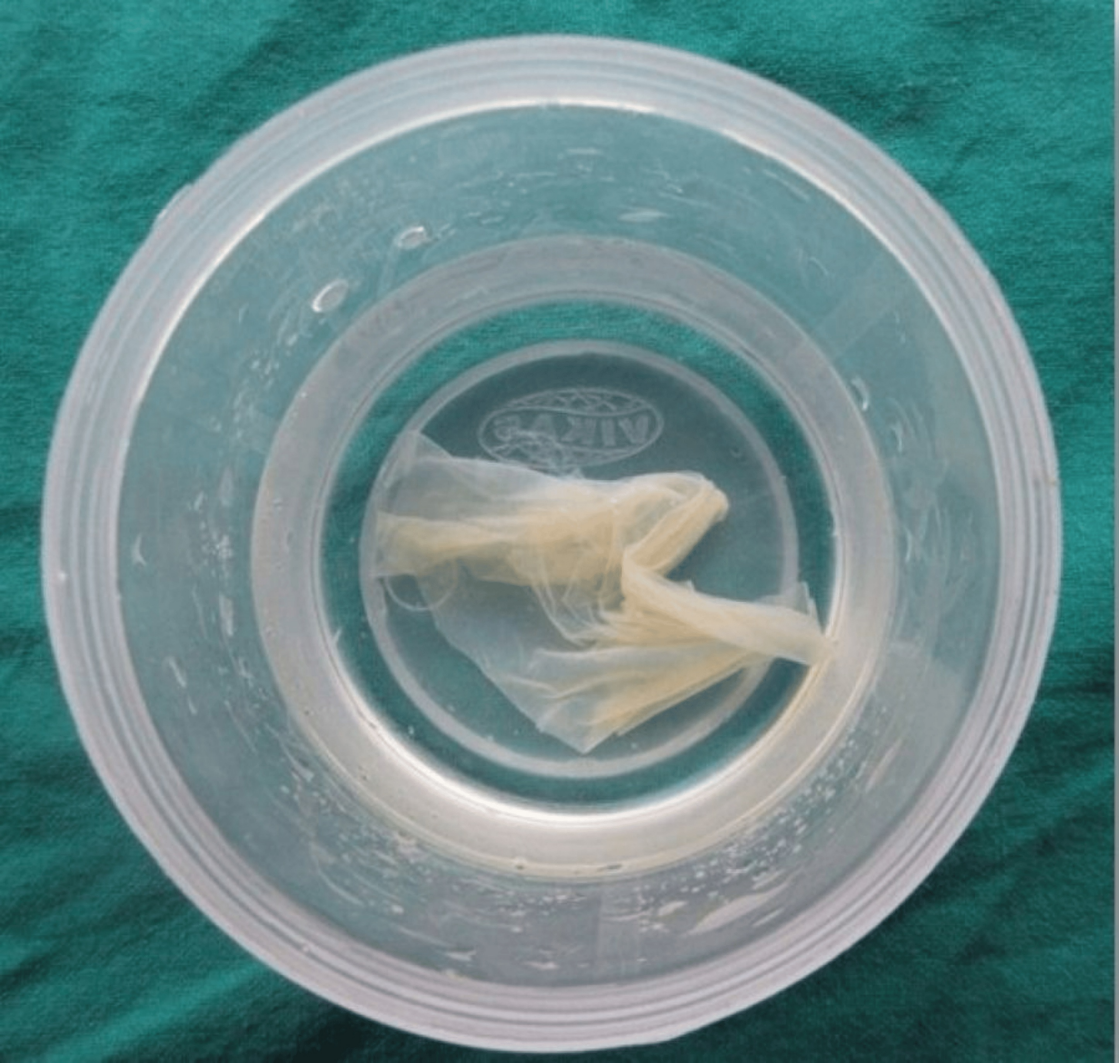
Amniotic membrane stored in glycerol

The amniotic membrane was freshly procured from the gynecology department under strict aseptic conditions (Figure 2). It was stored in glycerol to maintain structural integrity, reduce antigenicity, and provide antimicrobial preservation (Figure 3). To further enhance sterility, 1.5 lakh units of penicillin were added to the glycerol one day prior to surgery. This antibiotic supplementation aimed to minimize microbial contamination during storage and handling. The membrane was then stored in a refrigerator to maintain its biological properties and ensure safety. Immediately before placement, the membrane was thoroughly rinsed with sterile saline to remove excess glycerol and antibiotics, ensuring optimal biocompatibility with the recipient site.

Stage I: keratinized tissue augmentation

**Figure 4:**
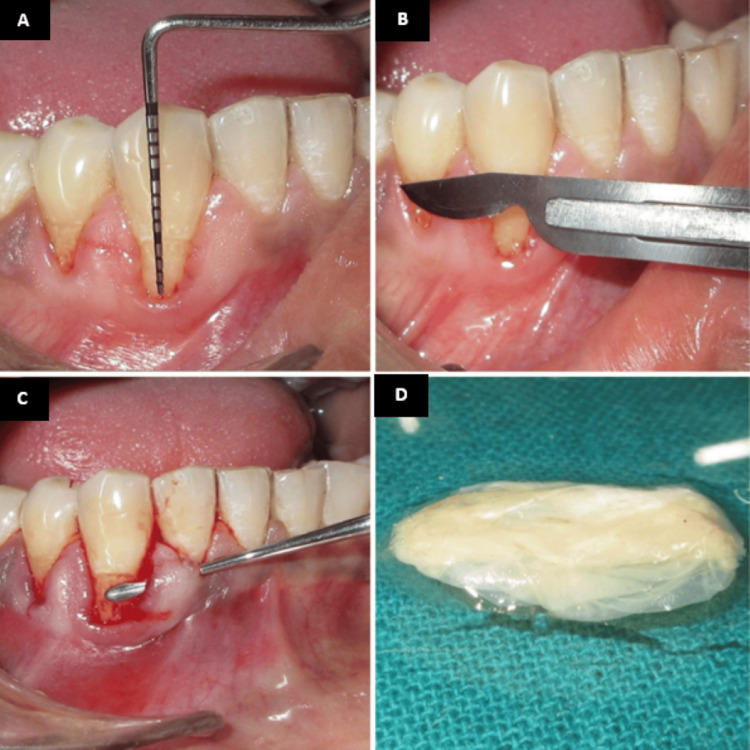
Tunneling with amniotic membrane. (A) Preoperative recession length measured using the UNC-15 probe. (B) Incision given using a 15c blade. (C) Tunneling done using tunneling knives. (D) Amniotic membrane UNC: University of North Carolina

**Figure 5:**
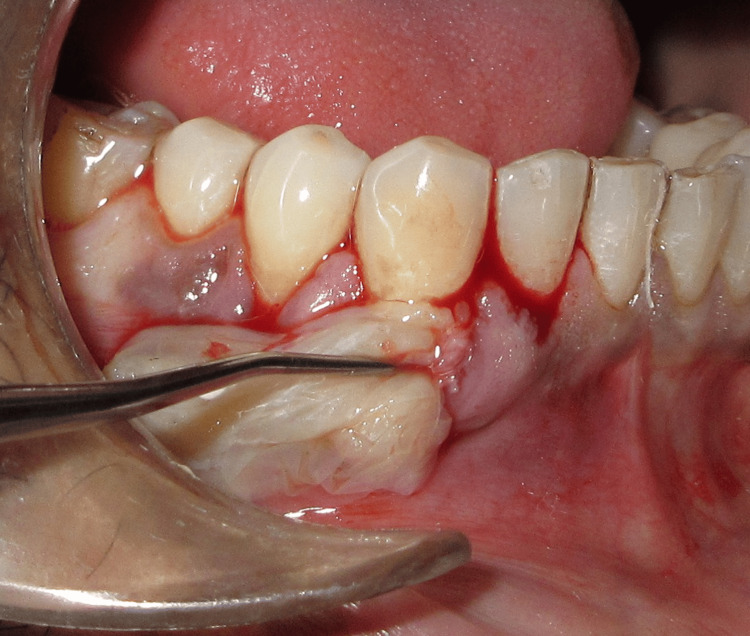
Amniotic membrane inserted inside the tunnel

Under local anesthesia, the recession length was measured and recorded using a University of North Carolina 15 periodontal probe (Figure 4A). A crevicular incision was given using a 15-c blade, and a minimally invasive tunneling technique was then employed to prepare the recipient site (Figure 4B). A partial-thickness tunnel was carefully created using specialized tunneling knives, extending beyond the recession defect while preserving the interdental papillae (Figure 4C). The amniotic membrane (Figure 4D) was gently inserted into the prepared tunnel (Figure 5) and positioned to adequately cover the area requiring augmentation.

**Figure 6:**
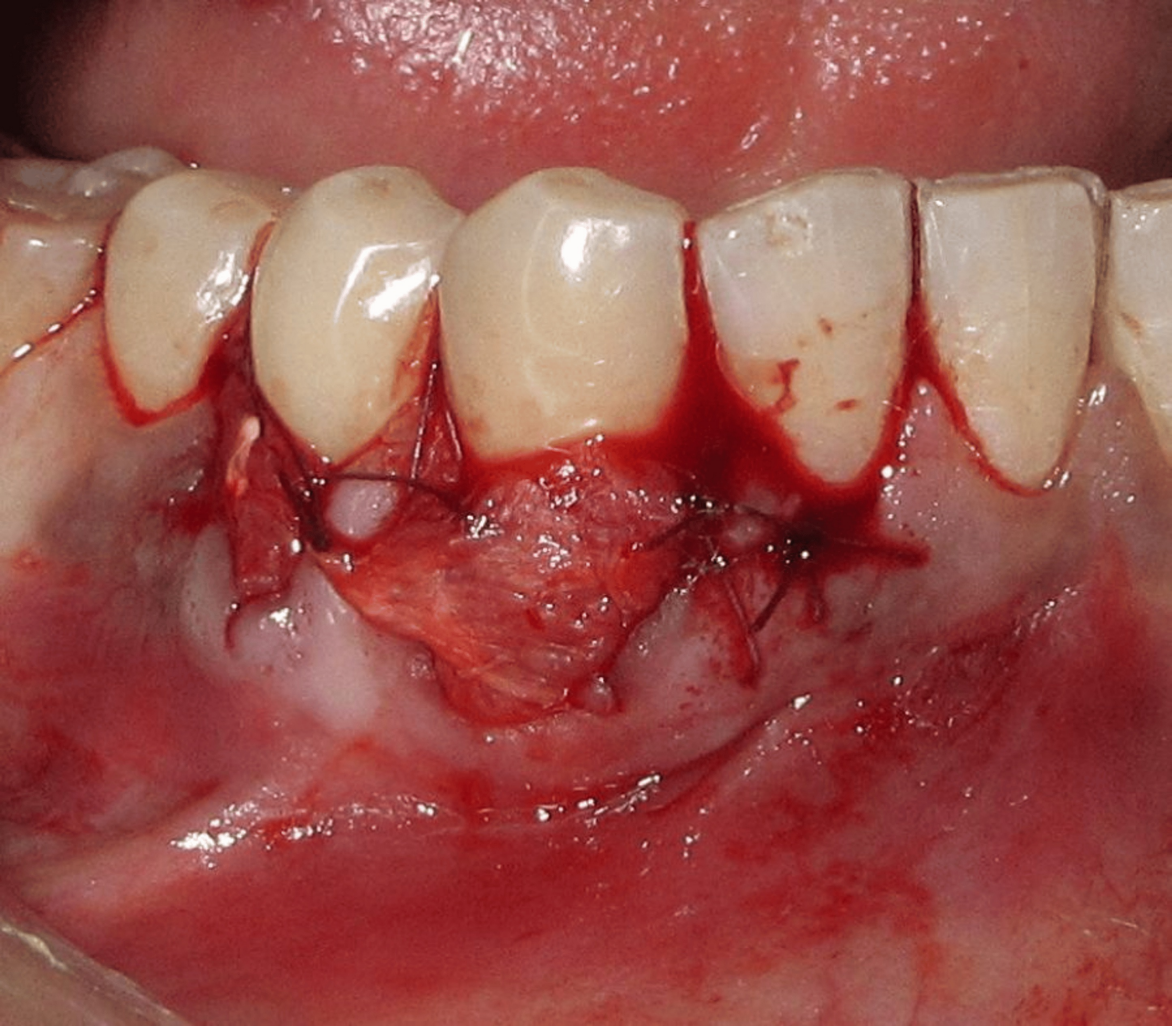
Flap coronally advanced and sutured

Following proper adaptation of the membrane, the flap was coronally advanced to the desired level and sutured to ensure stability and immobility (Figure 6).

**Figure 7:**
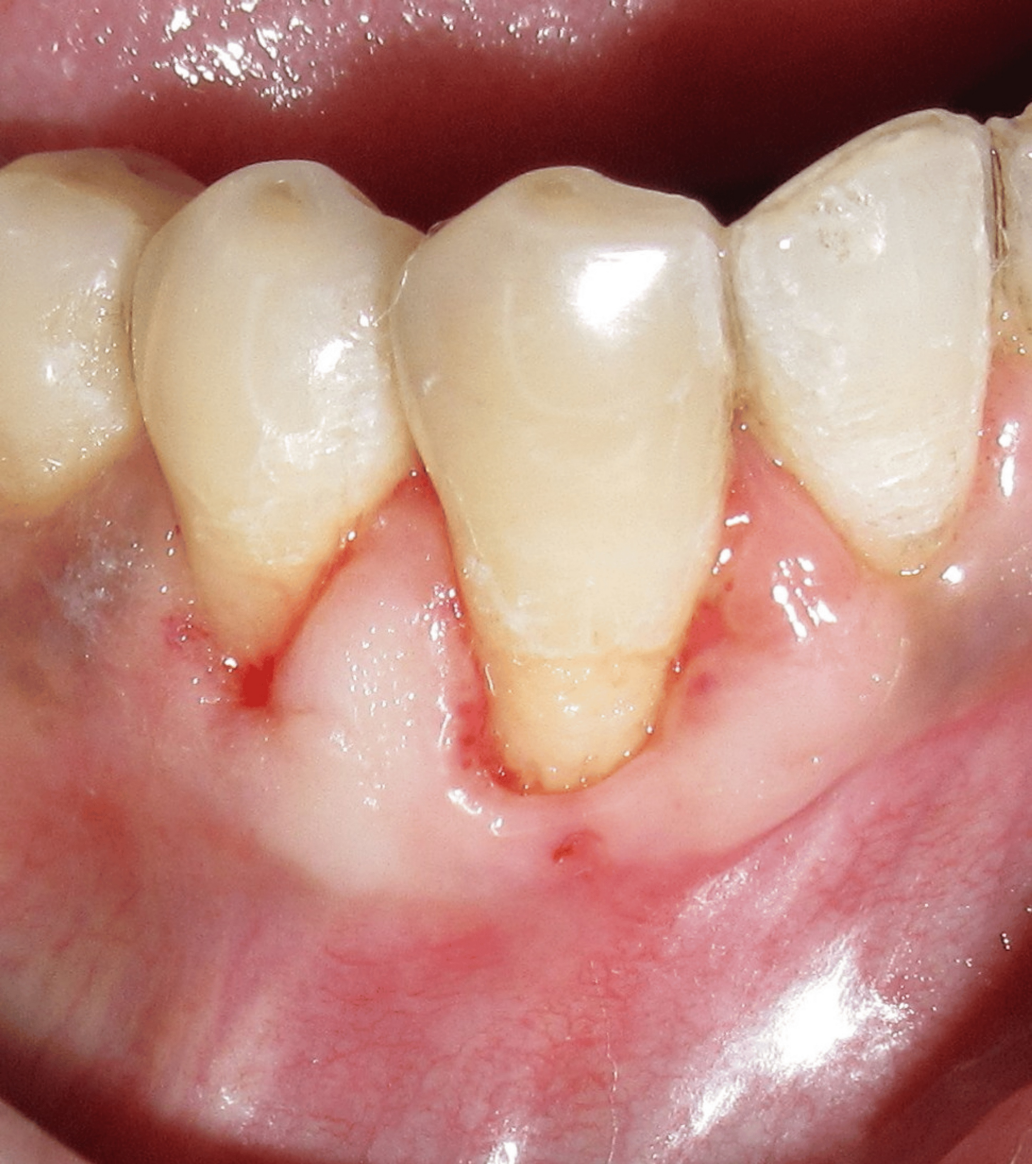
One-month postoperative image

Postoperative instructions were provided, and the site was allowed to heal for one month. Clinical evaluation at follow-up revealed a noticeable improvement in the gingival phenotype, which can be observed, establishing a stable and favorable recipient site for subsequent root coverage (Figure 7).

Stage II: lateral pedicle graft

**Figure 8:**
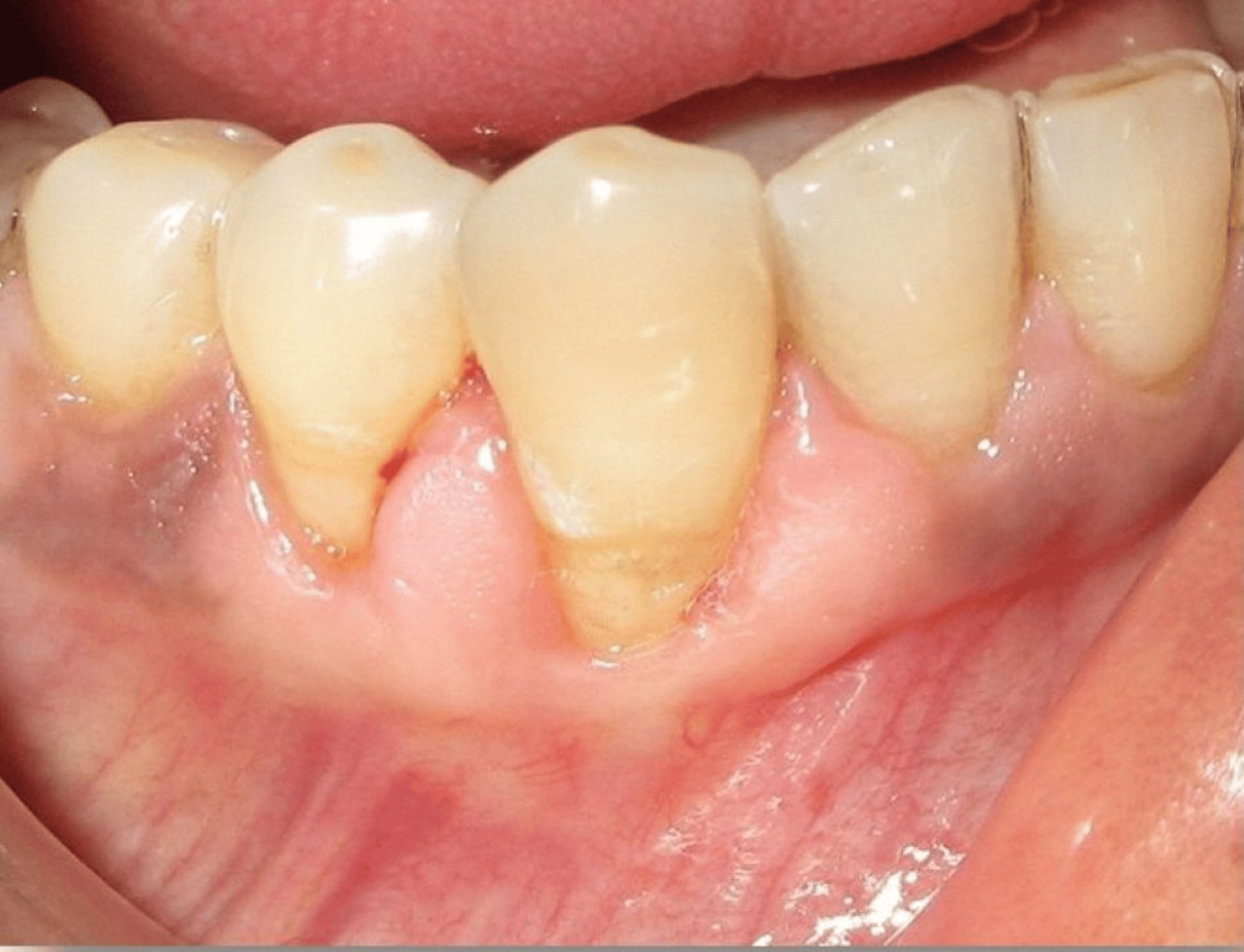
Preoperative view after one month of healing

After a healing period of one month (Figure 8), root coverage was performed using the lateral pedicle graft technique, which involves repositioning adjacent keratinized gingival tissue while maintaining its vascular supply.

**Figure 9:**
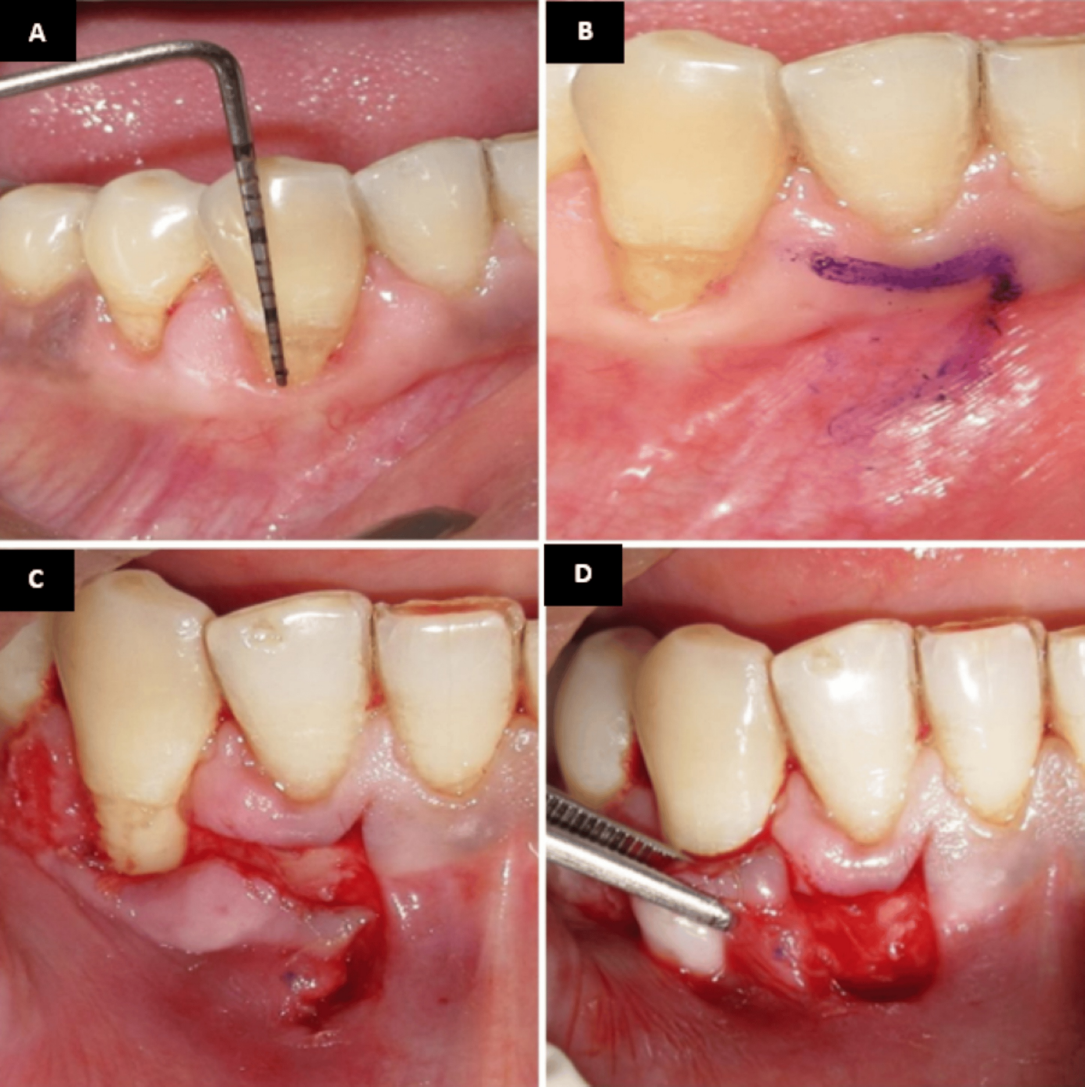
Lateral pedicle graft procedure. (A) Preoperative recession length measured using the UNC-15 probe. (B) Incision line marked using hematoxylin pencil. (C) Partial thickness flap raised. (D) Flap laterally displaced UNC: University of North Carolina

Under local anesthesia, the recession site was prepared, recession length was measured (Figure 9A), and a partial-thickness pedicle flap was outlined on the tooth (Figures 9B, 9C) adjacent to the defect, ensuring sufficient width and length of keratinized tissue at the donor site. Care was taken to preserve the marginal gingiva and interdental papillae of the donor tooth. A cut-back incision was made at the base (apical end) of the pedicle flap during a lateral pedicle graft to release tension and allow passive lateral movement of the flap to cover the recession defect, thereby maintaining an intact blood supply (Figure 9D).

**Figure 10:**
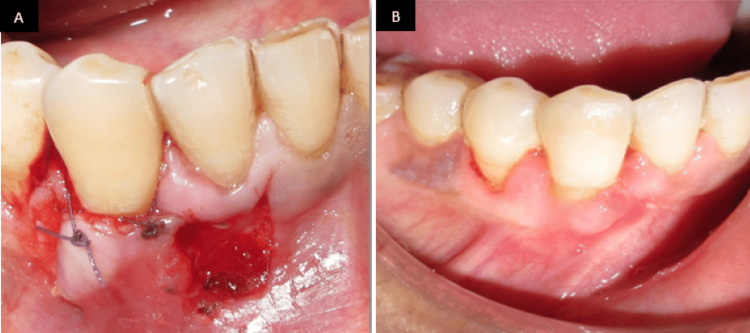
(A) Flap laterally displaced, coronally advanced, and sutured. (B) Three months postoperative view image

Following adequate flap mobility, it was laterally repositioned to completely cover the exposed root surface and adapted to the recipient site. The flap was stabilized using sutures placed without tension to ensure vascular stability and intimate tissue contact (Figure 10A). Postoperative healing was uneventful, and follow-up examination after three months revealed stable soft tissue architecture with harmonious gingival contours and satisfactory root coverage (Figure 10B).

## Discussion

Successful management of gingival recession depends on multiple anatomical and biological factors, among which the width and quality of keratinized tissue play a crucial role [6]. Although adequate tissue thickness contributes to flap stability, insufficient keratinized tissue width may compromise the predictability and long-term stability of root coverage procedures. In such situations, recipient site preparation becomes a key determinant of surgical success [7].

Amniotic membrane has gained increasing attention in periodontal therapy due to its unique biologic properties, including the presence of growth factors, promotion of epithelialization and angiogenesis, and modulation of inflammation [8]. Additionally, its low immunogenicity and antimicrobial potential make it a favorable biomaterial for soft tissue augmentation. In the present case, the use of amniotic membrane as a preliminary intervention resulted in a clinically appreciable increase in the width of keratinized tissue, thereby optimizing the recipient site for subsequent root coverage. Preservation of the membrane in glycerol, antibiotic supplementation, and refrigerated storage were employed to maintain sterility and biologic integrity prior to clinical use [9].

The tunneling technique used for membrane placement further enhanced the clinical outcome by minimizing surgical trauma, preserving interdental papillae, and maintaining vascularity [10]. This minimally invasive approach facilitates favorable wound healing and contributes to improved soft tissue stability.

The lateral pedicle graft is a well-established and predictable root coverage technique when adequate adjacent keratinized tissue is available [11]. Its advantage lies in the transfer of a vascularized flap, which promotes reliable healing and integration. In this case, performing the lateral pedicle graft after biologic site preparation allowed for tension-free flap adaptation and a stable soft-tissue architecture [12].

This case highlights the importance of a staged, biologically driven approach in managing gingival recession. Combining amniotic membrane-assisted keratinized tissue augmentation with a lateral pedicle graft may enhance surgical predictability, optimize soft tissue outcomes, and contribute to long-term periodontal stability.

## Conclusions

This case demonstrates that a staged, biologically driven approach can be effective in managing localized gingival recession associated with limited keratinized tissue width. Initial augmentation of the recipient site using an amniotic membrane provided a favorable soft tissue environment for subsequent root coverage. The use of a minimally invasive tunneling technique followed by a lateral pedicle graft allowed for stable flap adaptation and predictable healing. While the staged biologic preparation followed by a lateral pedicle graft resulted in satisfactory soft-tissue contours in this patient, conclusions are limited by the single-case design. Further controlled studies with objective measurements and longer follow-up are warranted to confirm the predictability of this approach.
